# Effectiveness of PitchSafe on Knowledge and Attitude of Baseball-Related Concussion

**DOI:** 10.7759/cureus.14732

**Published:** 2021-04-28

**Authors:** Chase C Labiste, Evan McElroy, Sotiris Chaniotakis, Nicolette Duong, Farzanna Haffizulla

**Affiliations:** 1 Internal Medicine, Nova Southeastern University Dr. Kiran C. Patel College of Allopathic Medicine, Fort Lauderdale, USA; 2 Internal Medicine, Nova Southeastern University Dr. Kiran C. Patel College of Osteopathic Medicine, Fort Lauderdale, USA

**Keywords:** youth sports, brain concussion, traumatic brain injury

## Abstract

Background

Traumatic brain injuries (TBIs), specifically concussions, affect many athletes and have dangerous immediate and long-term sequelae. Lack of awareness surrounding concussion impedes prevention, identification, and treatment. This study aims to assess parental knowledge and attitudes regarding concussions in youth baseball before and after administering an educational intervention called PitchSafe. PitchSafe is a short video that contains examples of baseball-related head injuries such as collisions, falls, and direct hits by baseballs; the signs and symptoms of a concussion; testimony from a former baseball player who sustained a TBI playing baseball; and a brochure detailing the potential signs, symptoms, and treatment plans associated with concussions.

Methodology

The Rosenbaum Concussion Knowledge and Attitudes Survey (RoCKAS) was used to assess three indices of parental understanding of concussion: Concussion Knowledge Index (CKI), Concussion Attitudes Index (CAI), and signs and symptoms of concussions (SS). The RoCKAS was administered at baseline and after the PitchSafe tool was administered. Parents were re-assessed one year following the initial distribution of survey materials utilizing the long-term follow-up (LTFU) RoCKAS. A paired sample t-test was conducted to compare the baseline, post-intervention (PI), and LTFU CKI, CAI, and SS among participants.

Results

The mean scores for CKI were 68% ± 12%, 76% ± 4%, and 76% ± 5% for baseline, PI, and LTFU, respectively. The mean scores for SS were 46% ± 20%, 62% ± 14%, and 64% ± 16% for baseline, PI, and LTFU, respectively. The mean scores for CAI were 87% ± 6%, 91% ± 5%, and 92% ± 4% for baseline, PI, and LTFU, respectively.

Conclusions

PitchSafe increased youth baseball parents’ long-term knowledge of concussions, ability to identify signs and symptoms of concussions, and may promote safer attitudes toward concussions. These findings support more widespread use of educational tools through social media and in clinical settings.

## Introduction

Traumatic brain injury (TBI), specifically concussion, is an unfortunate yet highly prevalent adverse medical event that impacts many athletes. The recent focus on concussion in the medical community has emerged because research has raised awareness of the potentially life-altering, long-term sequelae of these injuries, including cognitive decline, Alzheimer’s disease, depression, and chronic traumatic encephalopathy [1-5]. The Centers for Disease Control and Prevention (CDC) estimated that there were 2.87 million TBI-related emergency department visits, hospitalizations, and deaths in 2014, of which 837,000 occurred among children [6]. Concussion under-reporting is the disparity between the incidence of sport-related concussion (SRC) and those who seek medical services for SRC [7]. Concussion reporting rates are determined by complex multifactorial aspects, including knowledge, attitudes, socioeconomic status, geography, parental involvement, personal experience, and education level [8,9]. Neglecting medical attention for concussion is associated with poorer health outcomes [8-10].

An SRC is a TBI induced by biomechanical force to the head during participation in a sport [11]. These injuries commonly occur because of direct hits to the head, face, or neck; however, collisions, falls, and strikes with other individuals may also produce an impulsive force which can be transmitted to the head [11]. Recovery from the acute signs of concussion averages seven days; however, recent studies indicate that cognitive performance deficits persist past 14 days [12]. The long-term neurobehavioral sequelae of concussion are a subject of ongoing research. Emerging research is demonstrating long-term changes which occur as a result of concussion [13]. Due to the combination of acute symptoms and potentially long-term complications, it is crucial to reduce concussion risk and promote medical-seeking behavior when appropriate.

Much of the attention toward SRC has been focused on sports with higher rates of direct head impact exposure such as American football, ice hockey, and Australian football. Baseball is not traditionally thought of as a sport with significant direct head impact exposure. However, the pitching position in baseball may be particularly dangerous. Batters can hit a pitched baseball back to the pitcher at speeds faster than originally thrown [14]. Youth baseball players have among the lowest reported incidences of SRC 0.06/1,000 adverse events [15]. These findings may be the result of a deficit in the surveillance and identification of SRC in youth baseball. Athletic maneuvers such as sliding, diving, and collisions are thought to pose a low risk for concussions and therefore may lead to SRC that go unidentified and unreported [7].

Identifying the signs of SRC in youth baseball players early and accurately may prevent further injury and facilitate proper treatment. In youth baseball, the most numerous eyewitnesses to potential SRCs are the parents of youth athletes. HEADS UP is a series of educational initiatives, developed by CDC, which protects children and teens by raising awareness to improve prevention, recognition, and response to concussion and other serious brain injuries [16]. However, these programs are not designed to provide sport specific examples of bumps, blows, jolts, or hits to the head. Prior studies have demonstrated the effectiveness of the HEADS UP program to promote knowledge about concussion in high school athletes [17,18]. Despite this, these studies failed to show a significant change toward safer attitudes about concussion [17,18].

The present study is designed to determine whether the educational tool we developed, PitchSafe, positively impacts the long-term knowledge and attitudes of concussion in parents of youth baseball players. We created a baseball-specific educational tool tailored to scenarios that are likely to arise in this specific environment. The educational tool demonstrated sports-specific injuries, personal testimony of previously concussed athletes, and HEADS UP materials. The purpose of this study was to test the efficacy of this educational tool by measuring the improvement in concussion knowledge, attitudes, and the ability to recognize signs and symptoms of concussion. It was hypothesized that the sports-specific educational tool would produce a stable increase in knowledge about concussion and promote safer long-term attitudes from parents of youth baseball players.

## Materials and methods

Participants

This study was approved by the Institutional Review Board at Nova Southeastern University, and informed consent was obtained electronically. For the initial phase of this study, convenience sampling was done through social media platforms to recruit parents of youth baseball players for the initial and long-term intervention. The participants were required to write, understand, and speak English; had children under the age of 18 who played baseball at the time of the study; and had attended at least one of their child’s baseball games. Participants who did not meet the inclusion criteria, scored below 33% on the validity scale, did not complete the survey, or failed to view the video and pamphlet were excluded from the study. The Rosenbaum Concussion Knowledge and Attitudes Survey (RoCKAS) and PitchSafe tool were administered through Qualtrics® (Qualtrics International, Provo, Utah, USA). Patients were recruited through Facebook Groups and Pages. No names or identifying information about the participants were included in the surveys. Participants consented to the study by agreeing to participate in the survey.

PitchSafe tool

The PitchSafe tool comprises a brochure and a 1 minute and 55 seconds short video. The brochure addresses the following topics: What is a concussion? catastrophic re-injury, concussion management, and signs and symptoms of a concussion. The video and brochure were created using recommendations from the CDC and National Institutes of Health. The short video contains examples of baseball head injuries such as collisions, falls, and direct hits by baseballs; signs and symptoms of concussion; and testimony from a former baseball player who sustained a TBI playing baseball. The video provided a brief overview of the risks of concussions, baseball-specific examples of injuries, and personal testimony from a former youth baseball player who sustained a brain injury. The brochure provided easily understandable information about concussions in the form of bulleted lists and short sentences. It also provided more detailed explanation about the potential signs, symptoms, and treatment plans associated with concussions.

Instrument

The RoCKAS is a psychometrically analyzed and verified survey which assesses the knowledge and attitudes of individuals pertaining to concussion [19]. This survey has been utilized by other studies to reliably assess knowledge and attitudes about concussion since its publication [20-24]. The survey analyzes three indices: Concussion Knowledge Index (CKI), Concussion Attitude Index (CAI), and the Validity Scale (VS). Further analysis of the CKI yielded an additional index: Signs and Symptoms of Concussion (SS).

The CKI index comprises true/false, single-answer multiple choice and multiple-answer multiple choice questions. The responses assess participants’ knowledge of the causes and sequelae of concussion as well as signs and symptoms of a concussion [19]. Possible scores of the CKI range between 0 and 25; a higher score indicates a higher level of knowledge about concussion.

The SS score is a subsection of the CKI index which assesses a participants’ ability to identify signs and symptoms of a concussion. Possible scores of the SS range between 0 and 7. A higher score represents increased knowledge about the common signs and symptoms of concussion.

The CAI index assesses the safety of the participants’ decisions, and comprises Likert scales ranging from “Strongly Disagree” to “Strongly Agree” [19]. Higher scores represent safer attitudes about concussion. Possible scores of the CAI range between 15 and 75. A higher score represents safer attitudes about concussion.

The VS index assesses inconsistent effort or a lack of thoughtfulness [19]. These questions are not related to concussion and are answered at a rate of less than 5% incorrect [19]. Participants who score equal or less than one-third of VS index questions correct were excluded from the study.

Initial study

Social media posts through Facebook contained a recruitment message, consent document, and link to the Qualtrics® survey. Initial recruitment posts were distributed on Facebook Groups and Pages of Youth Baseball Leagues. Participants who met the inclusion criteria and consented to participate in the study were prompted to begin the baseline RoCKAS. After completing the baseline RoCKAS, parents were prompted to view the PitchSafe tool. After presentation of the educational intervention, participants indicated if they had viewed the video and brochure by multiple choice answers. Following the educational intervention, the participants were prompted to complete a post-intervention (PI) RoCKAS. The average response for the CKI, CAI, SS, and VS were calculated and compared using a paired t-test.

Long-term follow-up

Participants from the first phase of this research were recruited to the study one year following the initial distribution of the survey. Social media posts through Facebook contained a recruitment message, consent document, and link to the Qualtrics® survey. They were given a brief description about the project before being prompted to enter the survey. Participants who met the inclusion criteria and who participated in the initial study were instructed to complete the RoCKAS.

Statistical analysis

Based on similar studies assessing awareness of parents knowledge of concussions in football, a population proportion of 0.73 was used in calculating a statistically significant sample size [25]. A paired t-test was used to determine if a significant difference existed between the two mean scores obtained through the baseline and PI RoCKAS. The paired t-test was used in this study because it is a reliable test that assesses paired samples. The t-test demonstrated if there was a statistically significant change in mean survey responses after administration of the video and brochure. The paired sample t-test was used to assess participants mean change in knowledge, attitudes, and awareness of concussions that was obtained from the number of correct responses using the baseline and PI RoCKAS. The long-term follow-up (LTFU) average response for CKI, CAI, SS, and VS were calculated and compared using a t-test comparing initial study pre and post-PitchSafe education module. The software SPSS (IBM Corp., Armonk, NY) was used to statistically analyze the samples.

## Results

A total of 427 parents of youth baseball players were recruited to participate in the initial study. A total of 254 parents of youth baseball players were recruited to participate in the long-term study. Of the total participants in the initial study, 86 were excluded because they did not meet the inclusion criteria or scored less than 33% on the VS index. Of the total participants in the long-term study, 47 were excluded from because they did not meet the inclusion criteria, scored less than 33% on the VS index, or did not participate in the initial study.

Initial study results

The results of the study are summarized as percentages in each section of the RoCKAS in Table 1. There was a significant difference in the CKI scores for baseline RoCKAS (mean = 17.12, standard deviation [SD] = 3.090) and PI RoCKAS (mean = 19.10, SD = 1.127) conditions (T = 10.358, p < 0.001). A statistically significant difference in scores on SS was observed between baseline (mean = 3.68, SD = 1.623) and PI (mean = 4.95, SD = 1.303) conditions (T = 10.867, p < 0.001). The SD for SS was large because there were only eight possible signs and symptoms. Given the sample of the study, the means of SS were statistically significant despite overlapping SD. A statistically significant difference in scores on CAI was was observed between baseline (mean = 52.01, SD = 3.866) and PI (mean = 54.63, SD = 3.103) conditions (T = 8.749, p < 0.001).

**Table 1:** Results of survey expressed as percentage (mean ± SD) for CKI, SS, and CAI correct. PI: post-intervention; LTFU: long-term follow-up; CKI: Concussion Knowledge Index; SS: Signs and Symptoms; CAI: Concussion Attitude Index; SD: standard deviation

	Baseline	PI	LTFU
CKI score	68 ± 12	76 ± 4	76 ± 5
SS score	46 ± 20	62 ± 14	64 ± 16
CAI score	87 ± 6	91 ± 5	92 ± 4

Long-term follow-up results

The results of the follow-up survey (mean = 18.91, SD = 1.367) resulted in a significant difference between the baseline CKI and follow-up CKI (T = 7.878, p < 0.001), but there were no significant changes in the PI CKI and follow-up CKI (T = 1.763, p = 0.079). The results of the follow-up survey (mean = 5.12, SD = 1.265) resulted in a significant difference between the baseline SS and follow-up SS (T = 10.91, p < 0.001), but there were no significant changes in the PI SS and follow-up SS (T = 1.497, p = 0.135). The results of the follow up survey (mean = 55.26, SD = 2.534) resulted in a significant difference between the baseline CAI and follow-up CAI (T = 10.78, p < 0.001), and there was also a significant increase in follow-up CAI compared to PI CAI (T = 2.464, p = 0.014).

## Discussion

The aim of this study was to determine whether the PitchSafe educational tool positively impacted knowledge and attitudes about concussion. Programs such as HEADS UP, from the CDC, provide valuable information about concussion for parents [16]. However, these programs are not designed to provide sport-specific examples of bumps, blows, jolts, or hits to the head. Therefore, we created a baseball-specific educational tool to improve parental knowledge and attitudes surrounding concussion. The purpose of this study was to evaluate the efficacy of this educational tool by measuring the improvement in concussion knowledge (CKI), attitudes (CAS), and the ability to recognize signs and symptoms of concussion (SS) both immediately following the intervention and one-year PI. The results show significant improvements in all these domains immediately following the intervention. The improvements in CKI, SS, and CAI remained stable over the period of a year. From this we conclude that the parents who participated retained their knowledge or continued to investigate this topic on their own accord. The follow-up results did not significantly improve compared to the PI results which suggests that the parents may not have participated in other concussion education programs between intervention and follow-up.

The web-based design of this study reduced geographical selection bias and was cost-saving; however, the study had several design limitations. Participants completed the PI RoCKAS immediately following the completion of the educational tools. As such, the PI RoCKAS improvements may be subject to recency bias. Another limitation, common to many unsupervised web-based surveys, is the potential for participants to engage with others, receive help on questions, or search for answers [26].

Identifying signs and symptoms of a concussion is not only critical to accurate reporting but also to the child’s recovery. The fact that there was year-long retention of knowledge about the signs and symptoms of a concussion is promising and suggests that these parents may be more likely to recognize that their child needs medical care following an injury. The low baseline scores suggest that parents initially did not have a fundamental understanding of concussions. This finding supports the need for additional concussion education as better understanding may promote safer practices by parents. Previous studies have demonstrated that parents have difficulty in identifying a concussion in their children [27]. Recognition of concussion by parents is especially important as adolescents are more prone to the deleterious effects of sports concussions than adults, and adherence to concussion treatment in adolescents is generally associated with a faster recovery time [28].

Prior educational intervention strategies, using CDC resources such as HEADS UP, did not demonstrate significant changes in attitudes [17]. Significant changes toward safer attitudes (CAI) in the present study could be attributed to the sports-specific format of the educational materials. Incorporating baseball-specific types of injuries such as diving, direct baseball impact, and collision injuries may have increased the relevancy of the educational video and therefore increased appeal to the parents. Safer attitudes are correlated with increased reporting of suspected concussion and is associated with a safer parental attitude toward concussion for high school students [29]. The baseball-specific educational materials were effective at increasing parental CKI, CAI, and SS. Though this may lead to increased reporting, the results of our study do not indicate that directly. Further research is needed to elucidate how this education can translate into behavioral change.

## Conclusions

The findings from this study suggest that expansion of our educational tool, or a similar adaptation, to youth baseball leagues and physical examination could have a positive impact on the health of children participating in youth baseball. In addition, sports-specific educational tools for parents may be more effective at positively impacting concussion knowledge, attitudes, and symptom identification compared to general interventions. Future studies should specifically focus on other sports and explore whether sport-specific educational tools impact concussion reporting behaviors by parents.
